# Dihydromyricetin Enhances Intestinal Antioxidant Capacity of Growing-Finishing Pigs by Activating ERK/Nrf2/HO-1 Signaling Pathway

**DOI:** 10.3390/antiox11040704

**Published:** 2022-04-02

**Authors:** Chuan Wei, Xiaoling Chen, Daiwen Chen, Bing Yu, Ping Zheng, Jun He, Hong Chen, Hui Yan, Yuheng Luo, Zhiqing Huang

**Affiliations:** 1 Key Laboratory for Animal Disease-Resistance Nutrition of China Ministry of Education, Institute of Animal Nutrition, Sichuan Agricultural University, Chengdu 611130, China; 18438613981@163.com (C.W.); xlchen@sicau.edu.cn (X.C.); chendwz@sicau.edu.cn (D.C.); ybingtian@163.com (B.Y.); zpind05@163.com (P.Z.); hejun8067@163.com (J.H.); yan.hui@sicau.edu.cn (H.Y.); luoluo212@126.com (Y.L.); 2 College of Food Science, Sichuan Agricultural University, Ya’an 625014, China; chenhong945@sicau.edu.cn

**Keywords:** dihydromyricetin, ERK, antioxidant, IPEC-J2 cells

## Abstract

Oxidative stress is one of the main factors affecting animal health and reducing performance. The small intestine is the primary site of free-radical attacks. Dihydromyricetin (DHM) is a flavonoid compound with antioxidant, anti-inflammatory, and other biological activities, which is mainly extracted from Rattan tea. However, the effects of DHM on the intestinal antioxidant function of growing-finishing pigs and related mechanisms remain unclear. The aim of this study was to investigate the effect of dietary DHM supplementation on the intestinal antioxidant capacity of growing-finishing pigs and its mechanism. Our results show that dietary 0.03% DHM increased the activities of the total antioxidant capacity (T-AOC), catalase (CAT), and glutathione peroxidase (GSH-Px), decreased malondialdehyde (MDA) level, and upregulated protein expressions of HO-1, NQO1, nuclear Nrf2, and phospho-ERK (p-ERK) in the jejunum of growing-finishing pigs. Again, we found that 20 μmol/mL and 40 μmol/mL DHM treatment significantly upregulated the protein expression of HO-1 and promoted the nuclear translocation of Nrf2 and ERK phosphorylation in IPCE-J2 cells. ERK inhibitor PD98059 eliminated the DHM-induced upregulation of p-ERK, nuclear Nrf2, and HO-1. Our findings provided the first evidence that DHM enhanced the intestinal antioxidant capacity of growing-finishing pigs by activating the ERK/Nrf2/HO-1 signaling pathway.

## 1. Introduction

Oxidative stress is common in intensive pig production. Oxidative stress is defined as the result of an imbalance in reactive oxygen species (ROS) production and elimination in organisms [1]. ROS in small amounts are important intercellular signal transducers. However, when ROS production is excessive or the body’s antioxidant capacity is reduced, the excessive ROS cannot be removed in time, resulting in cellular structural damage. Under the modern breeding mode, pigs are subjected to a series of adverse stimuli from birth to market, such as environmental changes, heat stress, feed contamination, and pathogen invasion [2]. These factors can induce the body to produce excessive free radicals, causing oxidative damage in pigs.

The intestinal tract is not only the main site of nutrient digestion and absorption [3] but also the main organ attacked by free radicals [2]. Animal gastrointestinal mucosa is more vulnerable to oxidative damage due to its direct contact with ingested substances. The attack of free radicals on the intestinal tract can destroy the intestinal structure, resulting in a decrease in digestion and absorption ability, which, in turn, affects a pig’s production. ROS play an important role in oxidative stress-induced gastrointestinal mucosal injury [4]. ROS act on the target cell membrane, causing lipid peroxidation, cell membrane disintegration, and endothelial cell injury [5]. In addition, ROS can also lead to the release of pro-inflammatory factors, and the accumulation of pro-inflammatory factors can further promote the production of ROS and lead to intestinal tissue damage [6,7]. Oxidative stress not only affects gut health but also reduces productivity. Studies have found that lipid peroxidation in feed, such as soybean oil, can cause intestinal oxidative stress and reduce feed conversion rate and average daily gain [8]. Therefore, the intestinal antioxidant function is closely related to pig production. How to alleviate intestinal oxidative stress has become one of the main concerns of the pig industry.

Numerous studies have shown that some natural plant extracts have strong antioxidant activity and are a good choice for relieving oxidative stress in pigs [9,10,11,12]. Dihydromyricetin (DHM) is a flavonoid compound with antioxidant, anti-inflammatory, and other biological activities, which is mainly extracted from Rattan tea [13,14]. Numerous studies have shown that Rattan tea extract can promote animals’ production and improve body health [15,16]. The previous study of our group found that dietary DHM can enhance the activities of antioxidant enzymes in the muscle, serum, and liver of growing-finishing pigs [14] and mice [17]. However, the effects of DHM on the intestinal antioxidant capacity of growing-finishing pigs and related mechanisms remain unclear.

Nrf2 is the upstream activator of Heme oxygenase 1 (HO-1) [18]. Previous studies have shown that Nrf2 plays an important role in protecting against tissue and cell damage caused by oxidative stress. HO-1 is a phase II detoxifying enzyme, which plays an important role in maintaining the redox state and inhibiting inflammation [19,20]. MAPKs are the downstream effects of antioxidant responses [21,22]. The subfamilies of MAPKs include extracellular signal-regulated protein kinase (ERK), c-Jun N-terminal kinase (JNK), and p38 MAPK [23]. ERK2 can promote Nrf2 nuclear transfer and initiate antioxidant gene transcription [24]. Here, we explored the effects of DHM on the intestinal antioxidant capacity of growing-finishing pigs and its underlying molecular mechanism.

## 2. Materials and Methods

All animal procedures were approved by the Animal Care Advisory Committee of Sichuan Agricultural University and carried out using the Guidelines for Experimental Animal Care of Sichuan Agricultural University under permit No. YYS200528.

### 2.1. Animals and Experiment Design

Twenty-four crossbred (Duroc × Landrace × Yorkshire) barrows with an average body weight of 26.95 ± 0.26 kg were randomly divided into the control group (basal diet), 0.01, 0.03, and 0.05% DHM supplementation groups (*n* = 6). DHM was obtained from the Natural Field Biotechnology Co., Ltd. (Xi’an, China). The purity of DHM used in the pigs was ≥98%, and the purity of DHM used in the IPEC-J2 cells was ≥99.69%. The basal diet was formulated based on the NRC (2012) recommended nutrient requirements of 25–65 kg for 65 slaughter pigs. The dietary formula used is detailed in our previously published paper [14], and the nutrient level is a calculated value. DHM was added as follows. First, a small amount of limestone was premixed with DHM, and the amount of limestone was gradually increased until all limestone and DHM were mixed. Then, the soybean meal was added in the same way and then mixed with other feed ingredients to make the final diet. The feeding method was artificial feeding alone, and the feed was a powdered feed. All pigs were fed in independent cages (same size) and housed in a room. The experiment lasted for 15 weeks. All pigs were fed 3 times a day with free access to water. Feed intake was accurately recorded.

### 2.2. Sample Collection

At the end of the experiment, all the pigs were slaughtered by electronarcosis and exsanguination in a humane manner. Then, jejunum tissues and mucous membranes located in the same position were isolated and stored at −80 °C until analysis.

### 2.3. Cell Culture

The IPEC-J2 cell line was cultured in DMEM/F12 (Invitrogen, Carlsbad, CA, USA) supplemented with 10% fetal bovine serum (FBS, Gibco, Paisley, UK), 100 U/mL penicillin, and 100 mg/L streptomycin (Gibco) at 37 °C in a 5% carbon dioxide incubator. Cells were treated with different concentration of DHM for 36 h when the cells reached about 80% confluence. An ERK inhibitor PD98059 and cell nuclear protein extraction kit were purchased from Beyotime Biotechnology (Shanghai, China). DHM was purchased from Nature Field Biotechnology Co., Ltd. (Xi’an, China).

### 2.4. Western Blotting

Western blotting analysis was performed as previously described [25]. The total protein of jejunum mucosa was extracted with an RIPA lysis buffer (Beyotime, Shanghai, China) according to the instructions of the manufacturer. The protein concentration in jejunum mucosa was determined using a BCA protein assay kit (Pierce, Rockford, IL, USA) with a Nano-Drop ND 2000c Spectrophotometer. Proteins were isolated by gel electrophoresis and transferred to a PVDF membrane (Millipore, Eschborn, Germany) using a wet Trans-Blot system (Bio-Rad, Hercules, CA, USA). The PVDF membrane was sealed with 5% bovine serum albumin (Beyotime) at room temperature for 2 h and incubated overnight at 4 °C with the following primary antibodies: HO-1 (Proteintech, Chicago, USA, Cat. No. 66743-1-Ig), Nrf2 (Proteintech, Cat. No. 66504-1-Ig), NQO1 (Proteintech, Cat. No. 67240-1-Ig), ERK (Proteintech, Cat. No. 16443-1-AP), phospho-ERK (p-ERK) (Proteintech, Cat. No. 28733-1-AP), Histone (Beyotime, AF0009), and β-actin (Trans, Beijing, China, Cat. No. HC201) antibodies. The PVDF membrane was cleaned three times with TBST for 10 min each, incubated with the corresponding secondary antibody at room temperature for 2 h, and cleaned with TBST three times for 10 min each time. BeyoECL Plus (Beyotime) and the ChemiDoc XRS imaging system (Bio-Rad, California, USA) were used to collect protein signals, and Image Lab (Bio-Rad) was used to analyze protein expression.

### 2.5. Enzyme Activity and Malondialdehyde Content

The tissue homogenates were prepared as described above [26]. Approximately 0.1 g of the jejunum mucosa sample was homogenized in 0.9% saline and then centrifuged at 2500 r/min for 15 min at 4 °C. The supernatant was collected to determine antioxidant enzyme activities and MDA content. The activities of total antioxidant capacity (T-AOC), glutathione peroxidase (GSH-Px), total superoxide dismutase (T-SOD), catalase (CAT), and the content of malondialdehyde (MDA) in the jejunum mucosa were examined by commercial kits (Nanjing Jiancheng Bioengineering Institute, Nanjing, China) according to the manufacturer’s instructions. The total protein of the homogenate was measured by the Coomassie blue staining method using an assay kit purchased from the Nanjing Jiancheng Bioengineering Institute (Nanjing, China).

### 2.6. Statistical Analysis

Data were analyzed using the SPSS 27.0 statistics software (SPSS 27.0, Chicago, IL, USA) and expressed as mean ± standard error (SE). After checking for normal distribution and homogeneity of variance, the data were analyzed using a one-way analysis of variance (ANOVA) procedure, followed by Duncan’s multiple range test. *p* < 0.05 was considered statistically significant.

## 3. Results

### 3.1. Antioxidant Indicators in Jejunum Mucosa

The effect of DHM supplementation on the antioxidant indicators is presented in Table 1. Dietary DHM supplementation increased jejunum mucosa T-AOC and CAT activities (*p* < 0.05). Dietary supplementation with 0.01% DHM increased T-SOD activity (*p* < 0.05). Dietary supplementation with 0.03% DHM increased GSH-Px activity (*p* < 0.05). In addition, dietary supplementation with 0.03% DHM decreased MDA content (*p* < 0.05).

### 3.2. Antioxidant-Related Protein Expression in Jejunum Mucosa

**Figure 1:**
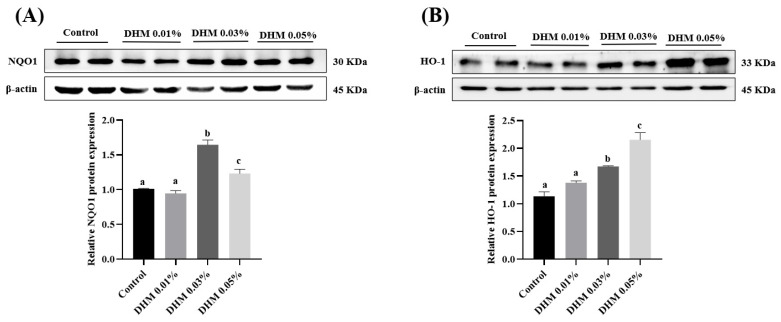

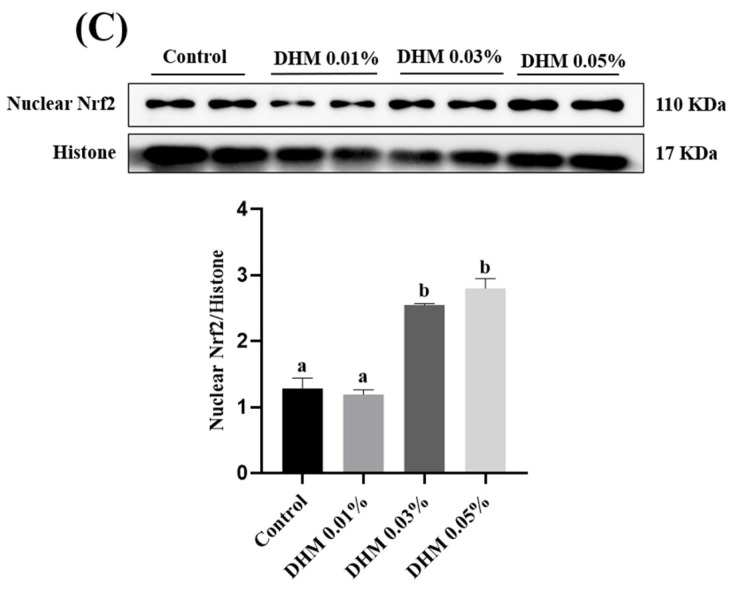
Effects of dietary DHM supplementation on antioxidant protein expression in the jejunum mucosa of growing-finishing pigs. (**A**) The protein level of NQO1. (**B**) The protein level of HO-1. (**C**) The protein levels of nuclear Nrf2. Data are expressed as the mean ± SE. Values with different superscript letters are significantly different (*p* < 0.05).

To explore the mechanism of DHM enhancing antioxidant capacity, we further examined the expression of an antioxidant-related protein. We focused on the Nrf2/HO-1 signaling pathway. As shown in Figure 1, dietary supplementation with 0.03% and 0.05% DHM significantly upregulated the protein expression of NQO1, HO-1, and nuclear Nrf2 in jejunum mucosa (*p* < 0.05).

### 3.3. Antioxidant-Related Protein Expression in IPEC-J2 Cells

**Figure 2:**
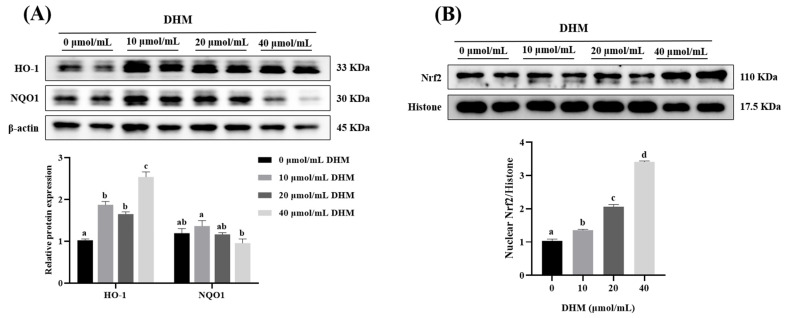
Effect of DHM on the protein expression of HO-1, NQO1, and nuclear Nrf2 in IPEC-J2 cells. IPEC-J2 cells were treated with DHM (0, 10, 20, 40 μmol/mL) for 36 h. (**A**) The protein levels of HO-1 and NQO1. (**B**) The protein level of nuclear Nrf2. Data are expressed as the mean ± SE. Values with different superscript letters are significantly different (*p* < 0.05).

As shown in Figure 2, after in vitro experiments, different concentrations (10, 20, 40 μmol/mL) of DHM significantly upregulated HO-1 protein expression and promoted Nrf2 nuclear translocation (*p* < 0.05). However, compared with the control group, DHM treatment had no significant effect on NQO1 protein expression (*p* > 0.05).

### 3.4. ERK Phosphorylation in Jejunum Mucosa and IPEC-J2 Cells

**Figure 3:**
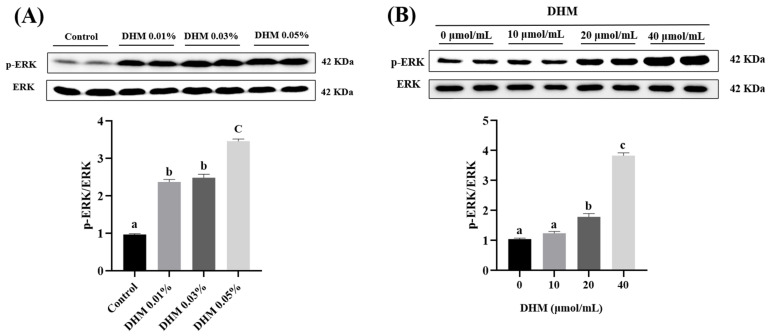
Effect of DHM on the protein expression of p-ERK. (**A**) The protein level of p-ERK in jejunum mucosa of growing-finishing pigs. (**B**) The protein level of p-ERK in IPEC-J2 cells. The protein expression of p-ERK was normalized to ERK. Data are expressed as the mean ± SE. Values with different superscript letters are significantly different (*p* < 0.05).

ERK is one of the upstream activators of Nrf2. We determined the p-ERK protein level. The results showed that DHM significantly increased the protein level of p-ERK in jejunum mucosa and IPEC-J2 cells (Figure 3) (*p* < 0.05).

### 3.5. DHM Activates the Nrf2/HO-1 Signaling Pathway by Activating the ERK Pathway

**Figure 4:**
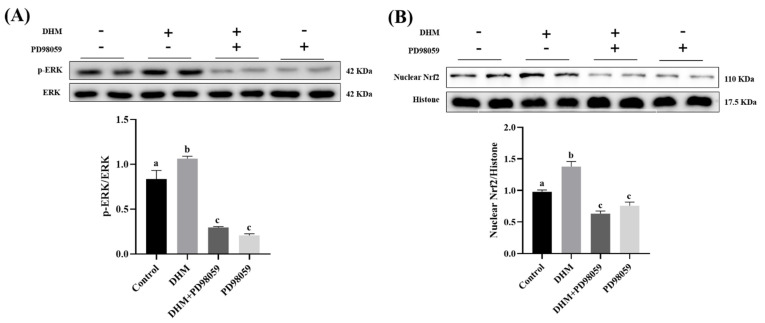

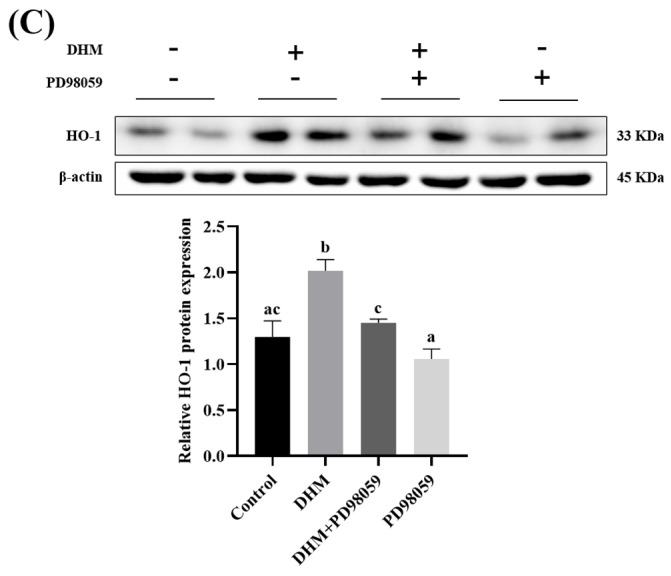
Effect of ERK inhibitor PD98059 on antioxidant-related protein expressions in IPEC-J2 cells. The IPEC-J2 cells were treated with DHM (20 μmol/mL) and PD98059 (10 μmol/mL) for 36 h. (**A**) The protein level of p-ERK. (**B**) The protein level of nuclear Nrf2. (**C**) The protein level of HO-1. Data are expressed as the mean ± SE. Values with different superscript letters are significantly different (*p* < 0.05).

To determine whether the ERK signaling pathway is involved in Nrf2 activation, the PD98059, a specific inhibitor of ERK, was used to inhibit the ERK signaling pathway in IPEC-J2 cells. Our results showed that treatment with 20 μmol/mL DHM significantly upregulated the protein expressions of p-ERK, nuclear Nrf2, and HO-1, which were eliminated by the PD98059 (Figure 4) (*p* < 0.05).

## 4. Discussion

The maintenance of intestinal health depends on the structural and functional integrity of intestinal epithelial cells [27]. Previous studies have shown that oxidative stress can lead to impaired intestinal barrier function [28,29]. Many environmental factors in pig production can induce oxidative stress in pigs, including stocking density, fighting, piggery hygiene, heat and cold stress, transportation stress, and *E. coli* infection [2]. These factors can affect the digestion and absorption of nutrients and cause oxidative stress in pigs [30,31]. Studies have shown that high-density feeding can destroy the antioxidant balance state of pigs and cause oxidative damage [32,33]. Therefore, the dietary supplementation of antioxidants to relieve oxidative stress is of great significance for animal production. Song et al. showed that L-cysteine could effectively reduce LPS-induced oxidative stress and intestinal inflammatory response in weaned piglets [29]. Silva-Guillen et al. found that dietary vitamin E supplementation could significantly enhance the total antioxidant capacity of weaned piglets [34]. Van et al. found that complex non-enzymatic antioxidants (vitamins A, C, and E, quercetin, organic Se, and GSH) could significantly reduce the oxidative stress induced by DON in piglets [35]. DHM has a variety of biological activities, including antioxidant, anti-inflammatory, antibacterial, etc. [14]. However, there are few studies on DHM in pigs. In this study, we investigated the effect of DHM on antioxidant capacity and the underlying molecular mechanisms in the jejunum mucosa of growing-finishing pigs and IPEC-J2 cells.

T-SOD is one of the important antioxidant enzymes in the body, which is mainly distributed in the cytoplasm and mitochondria. T-SOD plays an important role in maintaining low superoxide anion levels. GSH-Px plays an important role in preventing oxidative stress [36]. CAT protects cells from H_2_O_2_ toxicity by participating in a biodefense system [37]. Hua et al. showed that DHM improved endothelial dysfunction in diabetic mice via oxidative stress inhibition in a SIRT3-dependent manner [38]. Zhang et al. showed that DHM protected HUVECs from oxidative damage induced by sodium nitroprusside by activating the PI3K/Akt/FoxO3a signaling pathway [39]. Guo et al. found that DHM could significantly increase the antioxidant function of muscle in growing-finishing pigs [14]. Our results showed that dietary 0.03% DHM supplementation significantly increased the activities of T-AOC, CAT, and GSH-Px but reduced the MDA content in the jejunum of growing-finishing pigs. Our results are consistent with those of previous studies on pigs and rats.

Normally, Nrf2 binds to Keap-1, at which point Nrf2 is inactive. Upon oxidative stress, Nrf2 dissociates from Keap-1 and is transferred into the nucleus to promote the expression of HO-1 [40], NQO1 [41], and other antioxidant enzymes such as T-SOD [42], GSH-Px [43], and CAT [44], thus enhancing cellular defense against oxidative stress [45,46]. Previous studies showed that dietary DHM supplementation upregulated the protein expression of HO-1 and promoted Nrf2 nuclear translocation in muscle [14]. Here, our results showed that 0.03% and 0.05% DHM supplementation significantly upregulated HO-1 protein expression and promoted Nrf2 nuclear translocation in the jejunum mucosa. In IPEC-J2 cells, we also found that DHM treatment significantly upregulated HO-1 protein expression and promoted Nrf2 nuclear shift.

Previous studies have shown that various signaling pathways, such as PI3K/Akt and ERK, are involved in activating the Nrf2/HO-1 pathway [47,48]. Luo et al. found that DHM can promote the nuclear translocation of Nrf2 and HO-1 protein expression by activating the PI3K/Akt and ERK signaling pathways located in the human umbilical vein endothelial cells (HUVEC) [49]. In this study, we explored the effects of DHM on ERK phosphorylation in the jejunum mucosa of growing-finishing pigs. The results showed that DHM supplementation significantly increased the p-ERK protein level in jejunum mucosa. In IPEC-J2, we found that 20 μmol/mL and 40 μmol/mL DHM treatment significantly upregulated the protein expression of HO-1 and p-ERK and promoted the nuclear translocation of Nrf2. PD98059 is a specific inhibitor of ERK. Our results showed that PD98059 blocked ERK phosphorylation. In addition, HO-1 protein expression was significantly down-regulated, and Nrf2 nuclear translocation was inhibited when the ERK signaling pathway was blocked. This is consistent with the results of previous studies.

## 5. Conclusions

In conclusion, our findings provided the first evidence that DHM enhanced the intestinal antioxidant capacity of growing-finishing pigs by activating the ERK/Nrf2/HO-1 signaling pathway.

## Figures and Tables

**Table 1:** Effects of dietary DHM supplementation on antioxidant indicators in jejunum mucosal of growing-finishing pigs.

Items	Control	DHM 0.01%	DHM 0.03%	DHM 0.05%
T-AOC (U/mg prot)	0.37 ± 0.02 ^a^	0.53 ± 0.04 ^b^	0.74 ± 0.06 ^c^	0.70 ± 0.04 ^c^
CAT (U/mg prot)	24.56 ± 0.34 ^a^	36.42 ± 2.78 ^b^	33.70 ± 1.59 ^b^	34.15 ± 0.26 ^b^
T-SOD (U/mg prot)	9.02 ± 0.24 ^a^	14.09 ± 0.98 ^b^	10.91 ± 0.62 ^a^	10.55 ± 0.37 ^a^
GSH-Px (U/mg prot)	151.08 ± 7.31 ^a^	170.77 ± 8.58 ^a^	274.53 ± 19.64 ^b^	133.48 ± 6.52 ^a^
MDA (nmol/mg prot)	0.74 ± 0.05 ^a^	0.79 ± 0.01 ^a^	0.49 ± 0.06 ^b^	0.66 ± 0.03 ^a^

T-AOC: total antioxidant capacity; CAT: catalase; T-SOD: total superoxide dismutase; GSH-Px: glutathione peroxidase; MDA: malondialdehyde. Data are expressed as the mean ± SE (*n* = 6). Values within a row with different lowercase letters differ significantly at *p* < 0.05.

## Data Availability

Data presented in this study are presented in the article.
